# Supplementary material for: MeXpose—A Modular Imaging Pipeline for the Quantitative Assessment of Cellular Metal Bioaccumulation
Source: JACS Au. 2024 May 28;4(6):2197–210. doi: 10.1021/jacsau.4c00154 (PMC11200229; doi:10.1021/jacsau.4c00154)
Supplement: Supplementary file 1 — au4c00154_si_001.pdf [file au4c00154_si_001.pdf]

Supplementary Information

MeXpose - A modular imaging pipeline for the quantitative assessment of cellular metal bioaccumulation

Gabriel Braun[1,2¥], Martin Schaier[1,2¥], Paulina Werner[3], Sarah Theiner[1], Jürgen Zanghellini[1], Lukas Wisgrill[4,5], Nanna Fyhrquist[3], Gunda Koellensperger[1,5*]

[1] Institute of Analytical Chemistry, Faculty of Chemistry, University of Vienna, 1090 Vienna, Austria

[2] Vienna Doctoral School in Chemistry (DoSChem), University of Vienna, 1090 Vienna, Austria.

[3] Institute of Environmental Medicine, Karolinska Institutet, 17165 Solna, Sweden

[4] Division of Neonatology, Pediatric Intensive Care and Neuropediatrics, Department of Pediatrics and Adolescent Medicine, Comprehensive Center for Pediatrics, Medical University of Vienna, 1090 Vienna, Austria

[5] Exposome Austria, Research Infrastructure and National EIRENE Hub, 1090 Vienna, Austria

[¥] These authors contributed equally to the main findings of this manuscript.

* Corresponding author:

Gunda Koellensperger

Institute of Analytical Chemistry, 1090 Vienna, Austria

Tel: +43-1-4277-52303, Email: gunda.koellensperger@univie.ac.at

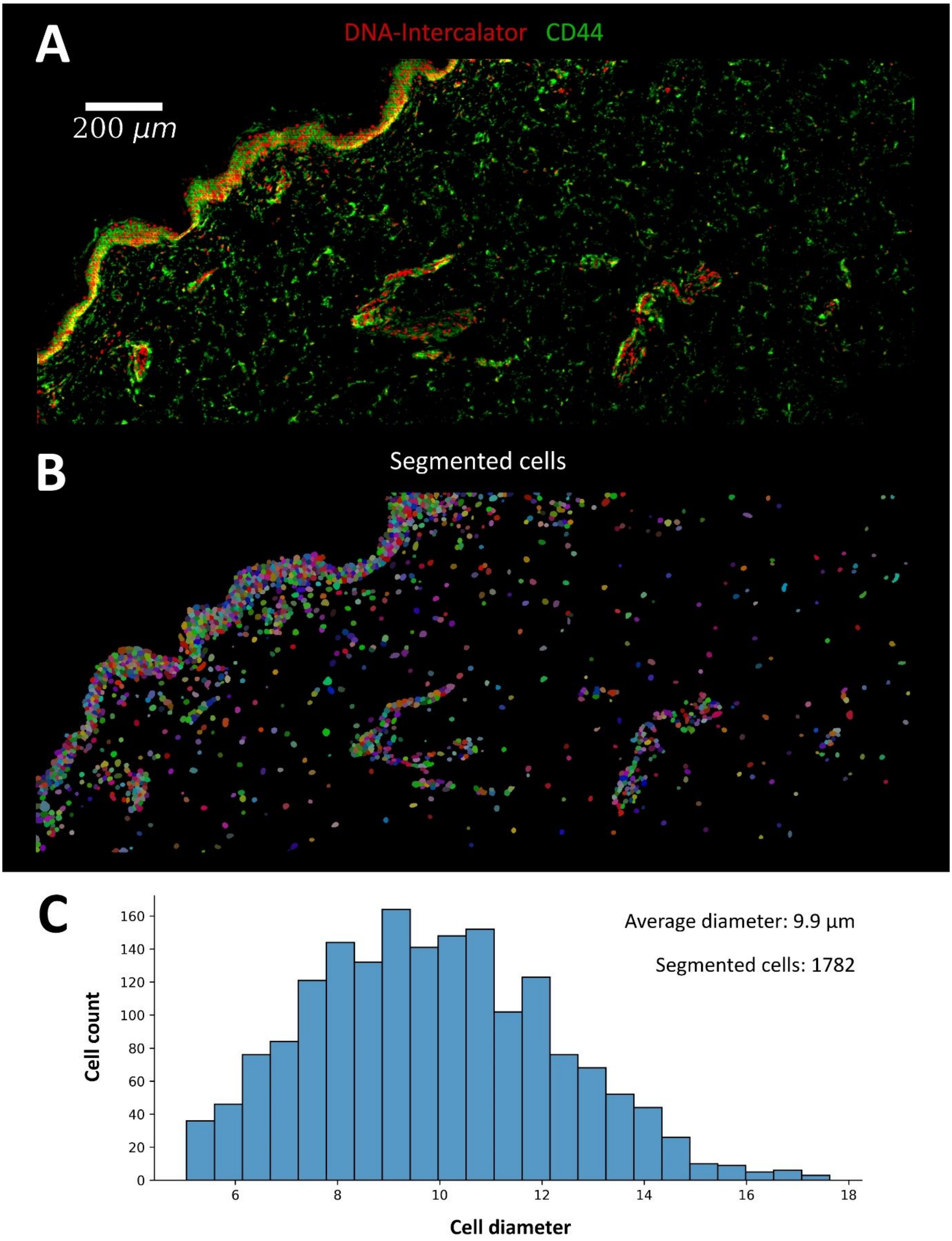

Figure S1: (A) Overlay images of cell membrane (CD44) and nuclei marker (DNA-Intercalator) in human skin as measured by LA-ICP-TOF-MS (1µm spatial resolution, 200 Hz ablation speed). These channels were used for segmentation following stacking and pre-processing. (B) Map of cellular objects (multidimensional data with

characterized area and intensity). (C) Size distribution of the cellular objects. A total of 1782 cells were segmented in the image with an average diameter of 10 µm.

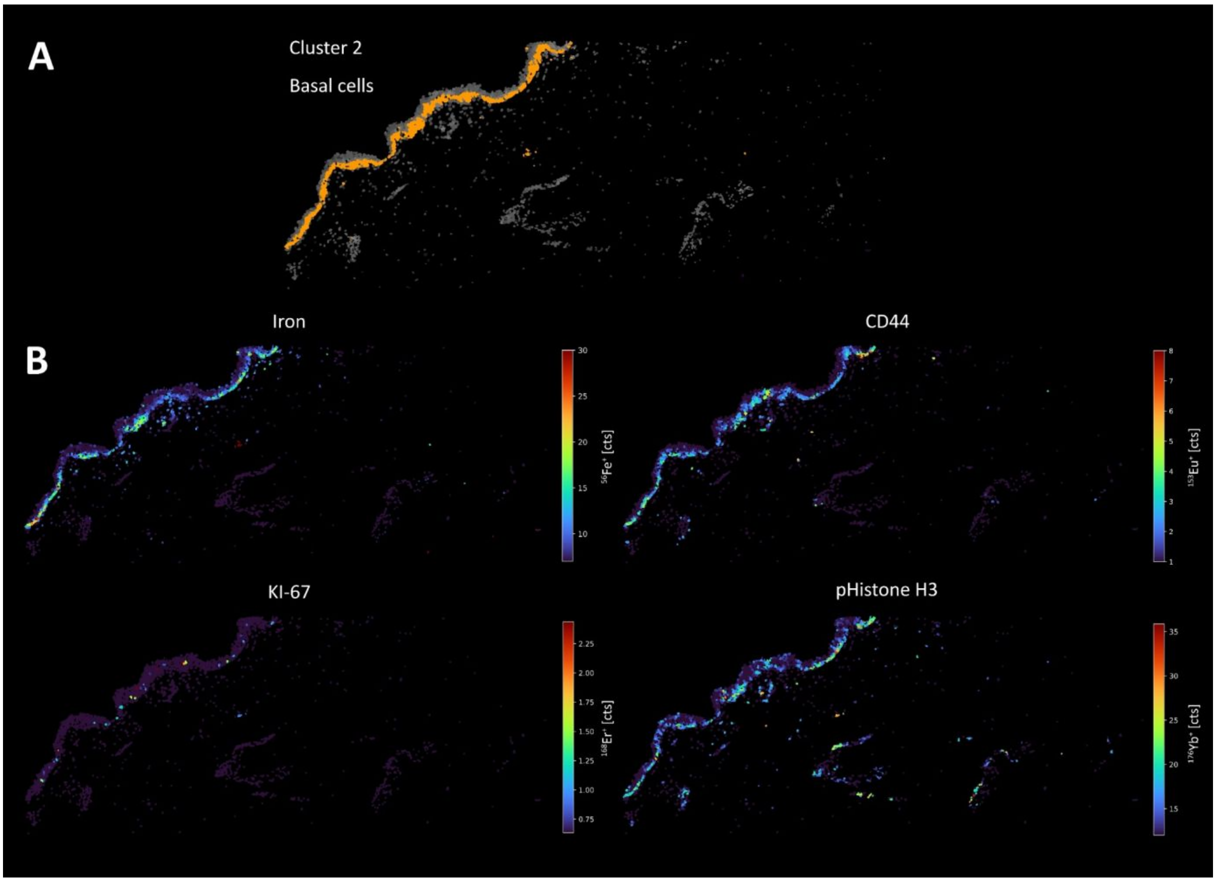

Figure S2: (A) Visualisation of cluster 2 (basal cells) within all of the segmented cells. (B) Fe showed a distribution characteristic of basal cells and was therefore helpful in phenotyping cluster 2, in addition to CD44, KI-67 and pHistone H3.

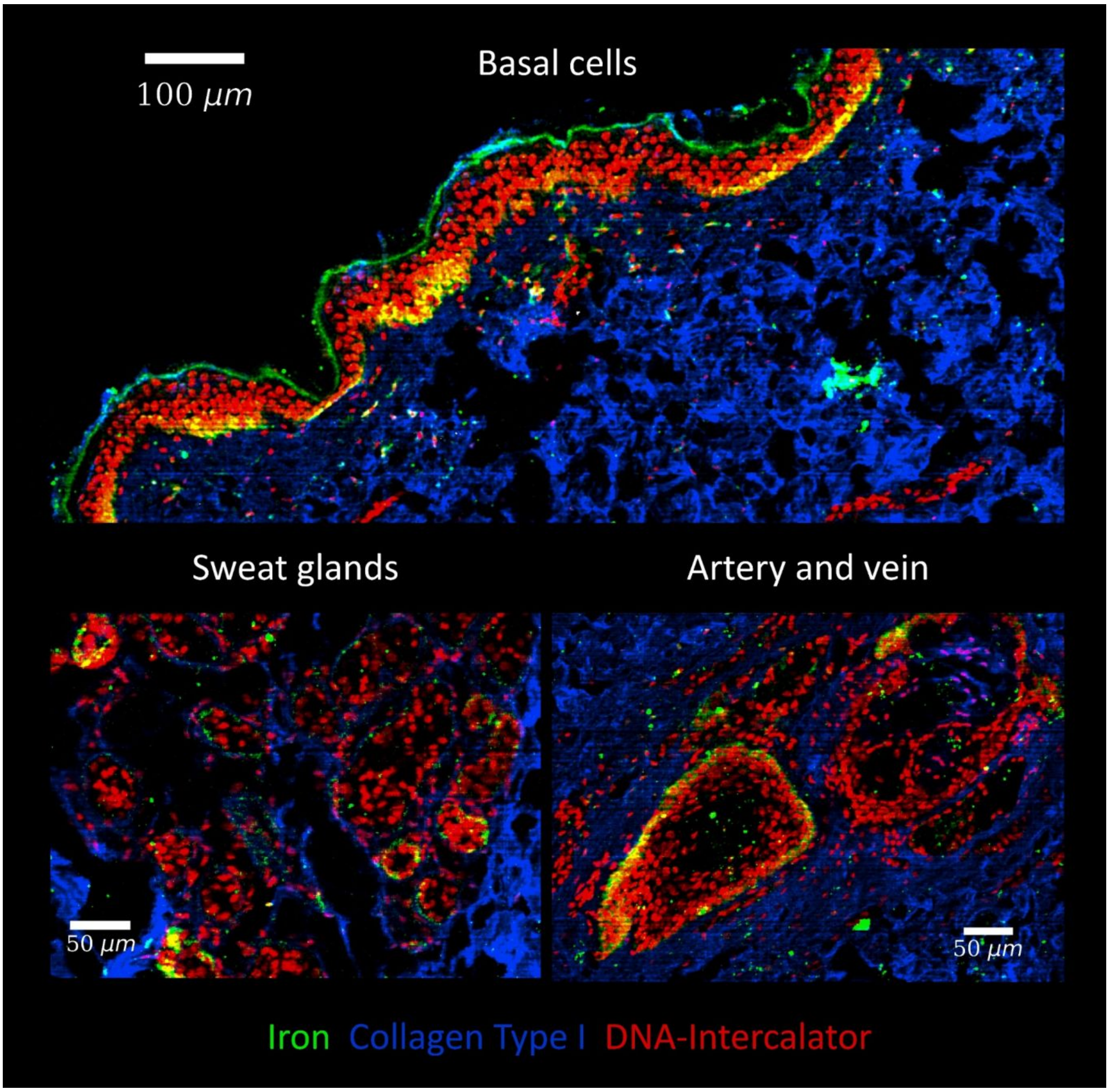

Figure S3: Signal overlay of Fe (green), collagen type I (blue) and DNA-intercalator (red) from different skin regions. Fe shows characteristic distributions in structural features of the skin such as basal cells, sweat glands or arteries even after FFPE treatment of the skin, which can improve phenotype clustering.

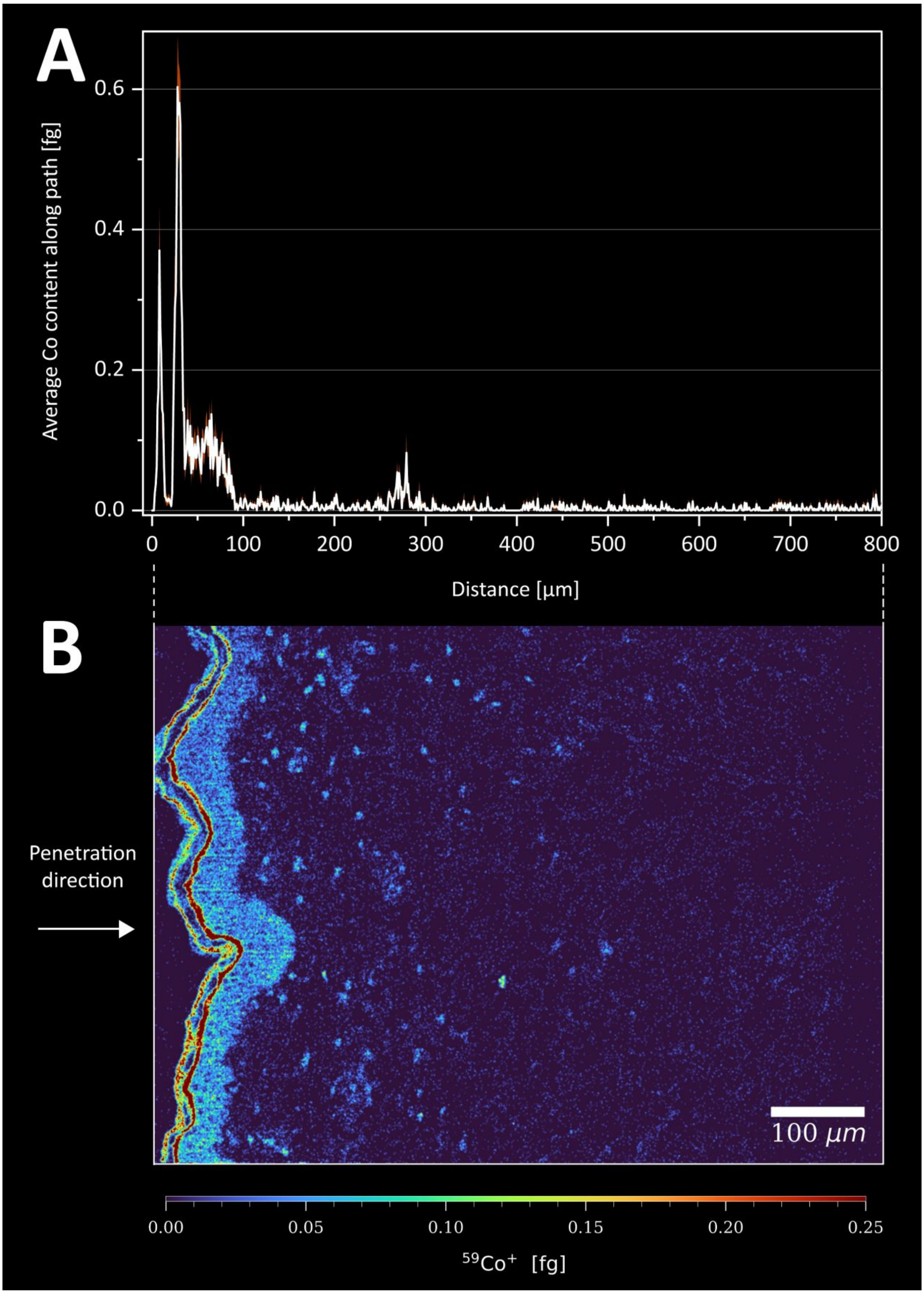

Figure S4: (A) Average amount of Co along a path plotted against the distance in the tissue. (B) shows the section on which the plot is based (LA-ICP-TOFMS analysis, 1 µm pixel size, 200 Hz pixel acquisition rate). The whole area has been integrated from left to right. The permeation mainly occurs 100 µm into the skin, i.e. into the epidermis. Only a small fraction of the Co is able to penetrate further into the dermis.

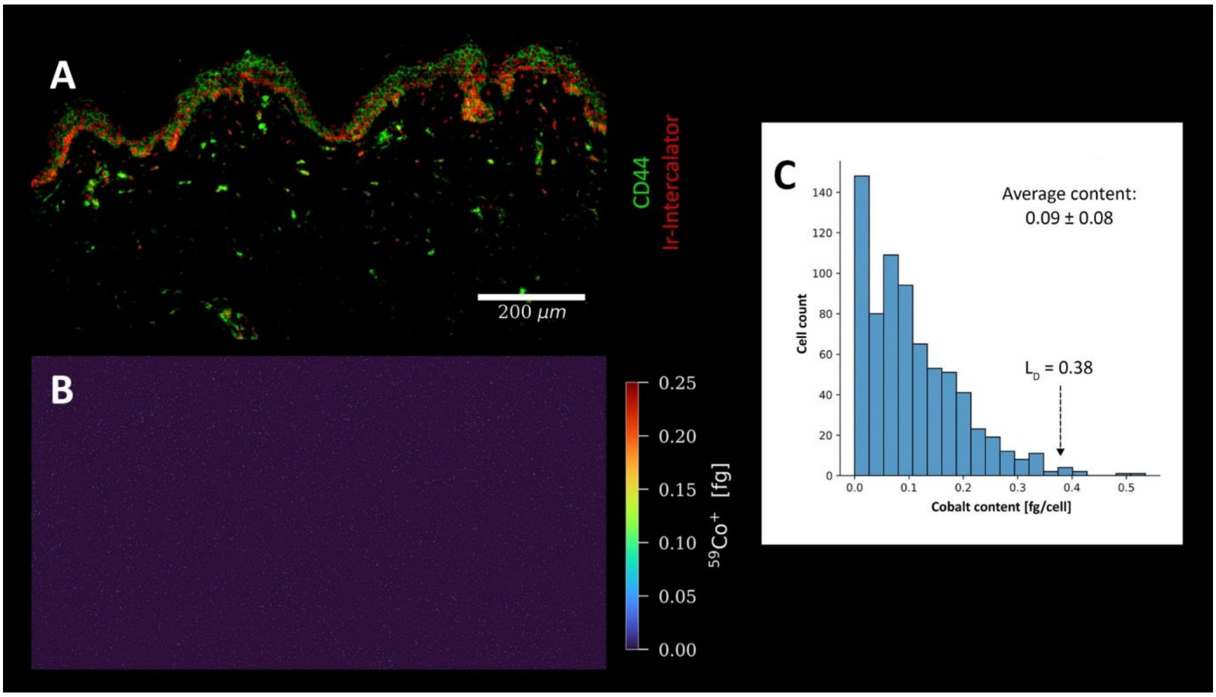

Figure S5: (A) Signal overlay of CD44 (green) and DNA intercalator (red) to visualise cells in a control sample (tagged with metal-conjugated antibodies, incubation with H_2O). The Co distribution in (B) shows that there is no measurable amount of Co in the tissue. There is only a background noise spread over the whole image. (C) shows the histogram for segmented cells with an average content of 0.1 fg. The LOD obtained is higher than 99% of the cells present in the control sample.

Null hypothesis using Mann-Whitney U test (Figure S6)

The single cell Co accumulation, obtained for each cell cluster, respectively (Fig. 6E), was tested for significant differences by statistical analysis. By using the Mann-Whitney U test a p-value matrix was generated allowing pair-wise comparison (Fig. S6). Cluster 0, characterised by the highest cell number, showed a notably low Co amount with an average of 1.2 fg per cell. There were no significant differences between clusters 3, 4 and 6, with mean contents of 2.1-2.2 fg per cell. Similarly, with an average of 2.8 fg per cell, clusters 1 and 5 showed considerable similarity. Notably, cluster 2 showed significant differences from all clusters (including a remarkable p-value of 4.22E-91 for cluster 0) and stood out with 3.8 fg per cell.

	Cluster 0	Cluster 1	Cluster 2	Cluster 3	Cluster 4	Cluster 5	Cluster 6
**Cluster 0**	1	4.07E-74	4.22E-91	4.29E-19	6.96E-22	2.05E-21	1.53E-11
**Cluster 1**	4.07E-74	1	7.46E-15	9.05E-16	2.78E-10	1.66E-01	1.93E-09
**Cluster 2**	4.22E-91	7.46E-15	1	7.38E-33	8.96E-25	1.47E-07	4.97E-18
**Cluster 3**	4.29E-19	9.05E-16	7.38E-33	1	1.35E-01	2.40E-04	8.46E-01
**Cluster 4**	6.96E-22	2.78E-10	8.96E-25	1.35E-01	1	7.23E-03	2.47E-01
**Cluster 5**	2.05E-21	1.66E-01	1.47E-07	2.40E-04	7.23E-03	1	2.14E-03
**Cluster 6**	1.53E-11	1.93E-09	4.97E-18	8.46E-01	2.47E-01	2.14E-03	1

Figure S6: Significant differences between clusters were assessed using p-values determined by the Mann-Whitney U-test. Using $\alpha = 0.05$ as the significance level, significant values (<0.05) were highlighted green and non-significant values (>0.05) red.

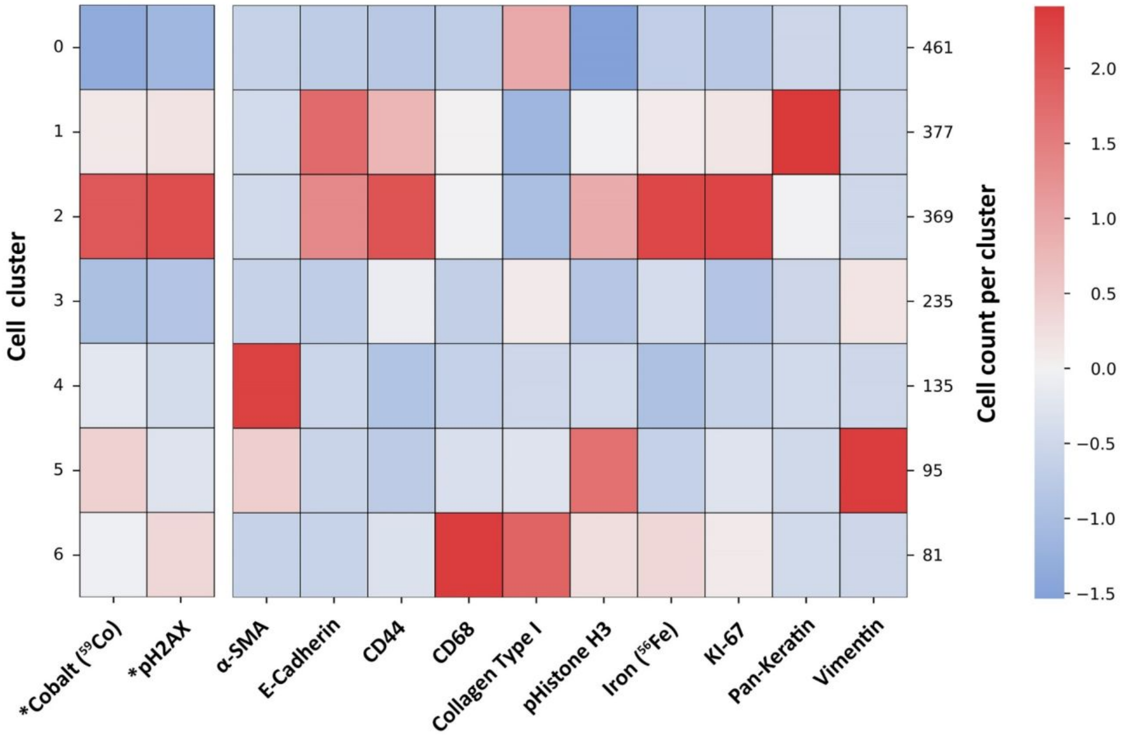

Figure S7: Heat map plotting Co and pH_2AX along the heat map of Fig. 4. As these read-outs are not characteristic of the phenotypes, they have not been included in the clustering itself. The high intensity of Co and DNA damage in cluster 2 can be clearly seen.

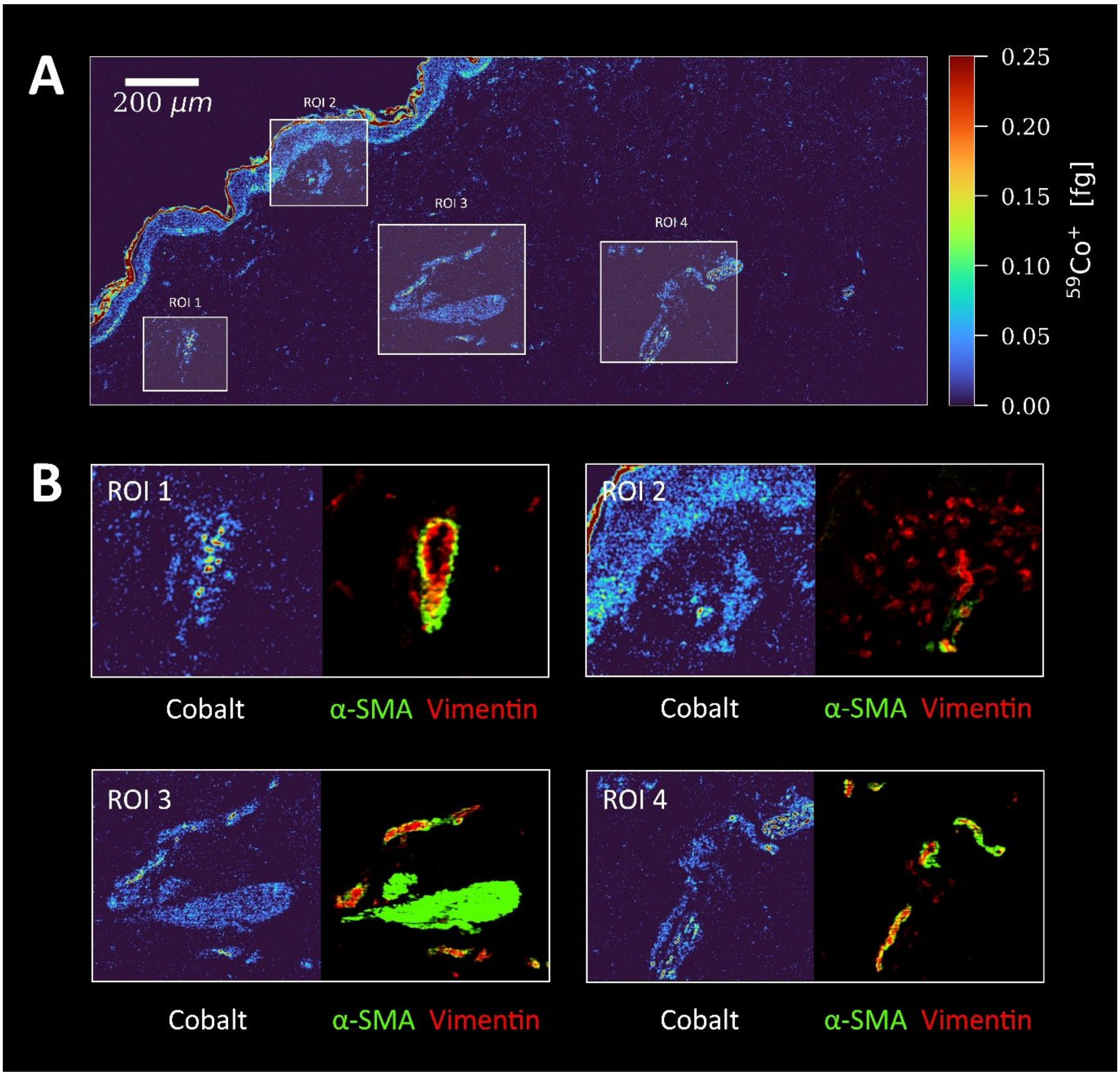

Figure S8: (A) Quantitative imaging of Co in human skin (LA-ICP-TOFMS analysis, 1 µm pixel size, 200 Hz pixel acquisition rate). Regions of interest (ROI) for blood vessels are outlined in the tissue section. (B) Co distribution compared with the location of blood vessels visualized by a-SMA (green) and vimentin (red). Increased accumulation in blood vessels is indicated.

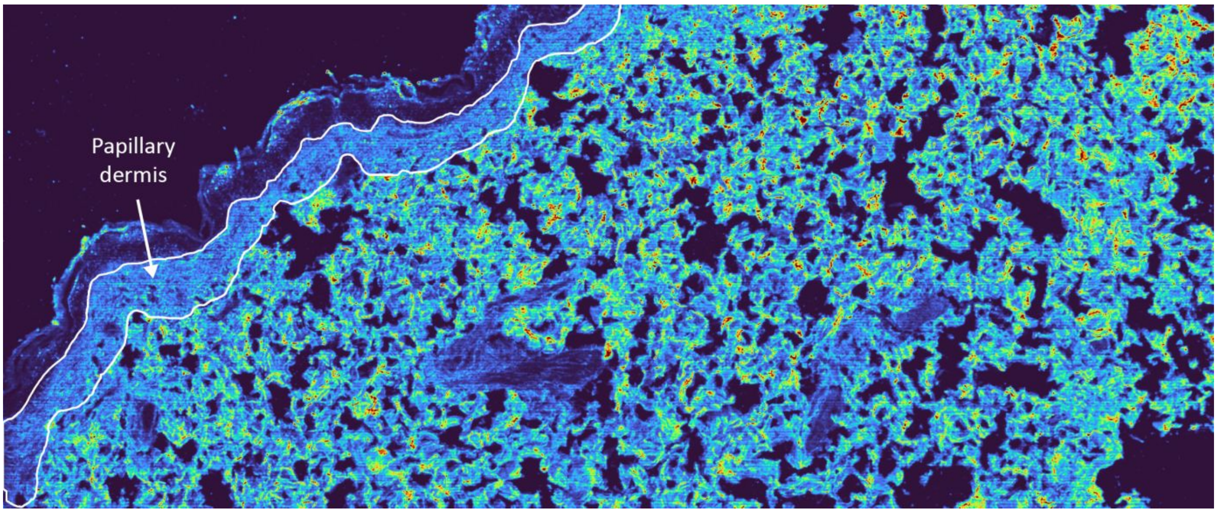

Figure S9: Signal intensity map of collagen type I (^{169}Tm) for human skin (LA-ICP-TOFMS analysis, 1 µm pixel size, 200 Hz pixel acquisition rate). Papillary dermis (outlined in white) can be distinguished from reticular dermis by collagen expression. While collagen fibers in the papillary dermis are thin and loosely arranged, those in the reticular dermis are thicker and densely packed.

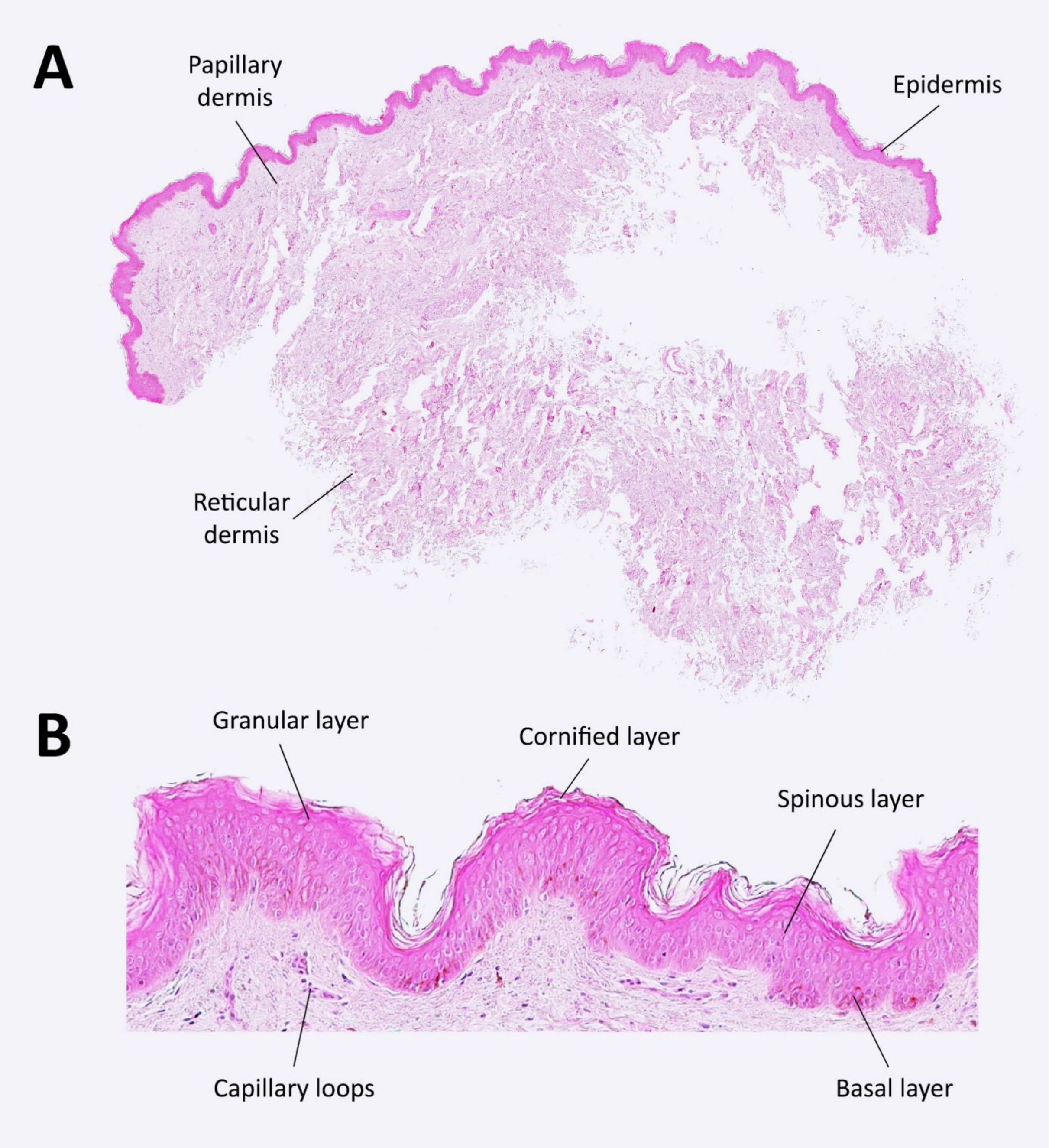

Figure S10: (A) Tissue section of NativeSkin stained with hematoxylin and eosin (H&E). The epidermis and papillary dermis were consistently observed in their entirety, although there were instances of incomplete reticular dermis due to the sectioning process. (B) provides a close-up of the epidermis for detailed examination.

Table S1: List of metal-conjugated antibodies

Antibody target	Clone	Metal tag	Catalog number	Target
**α-SMA**	1A4	^{141}Pr	3141017D	Smooth muscle cells
**Vimentin**	D21H3	^{143}Nd	3143027D	Mesenchymal cells, fibroblasts
**Pan-Keratin**	C11	^{148}Nd	3148020D	Cornified layer/ keratinocytes
**CD44**	IM7	^{153}Eu	3153029D	Hyaluronic acid receptor /intercellular adhesion
**E-Cadherin**	24E10	^{158}Gd	3158029D	Epithelial cell membrane
**Collagen Type I**	Polyclonal	^{169}Tm	3169023D	Dermal collagen fibers
**CD68**	KP1	^{159}Tb	3159035D	Macrophages
**pH2AX**	S139	^{165}Ho	3165036D	DNA damage
**KI-67**	B56	^{168}Er	3168022D	Proliferation
**pHistone H3**	HTA28	^{176}Yb	3176024D	Mitosis

Table S2: LA-ICP-TOFMS parameters

**ICP-TOFMS**	
RF Power [W]	1440
Sampling depth [mm]	3.5
Cone materials	Ni
Plasma gas flow [L min^{-1}]	14
Auxiliary gas flow [L min^{-1}]	0.80
Nebulizer gas flow [L min^{-1}]	1.06
Measurement mode	Collision cell technology (CCT)
CCT gas	93% He *(v/v)*, 7% H_2 *(v/v)*
CCT gas flow [mL min^{-1}]	4.2
m/z range	14-256
**Laser ablation**	
Spot size	2 µm (circular)
Interspacing	1 µm
Repetition rate	200 Hz
Dosage	2
Fluence	1.0 J cm^{-2}
